# Supplementary material for: Plant-Derived Smoke Affects Biochemical Mechanism on Plant Growth and Seed Germination
Source: Int J Mol Sci. 2020 Oct 20;21(20):7760. doi: 10.3390/ijms21207760 (PMC7588921; doi:10.3390/ijms21207760)
Supplement: Supplementary file 1 [file ijms-21-07760-s001.pdf]

Supplementary materials

Table S1. Effect of plant-derived smoke on germination and post-germination responses of various plants.

Experimental plant species	Major findings	References
Smoke solution for application		
Basterheide	Seed germination increased	de Lange and Boucher, 1990
Fire lilly	Flowering increased	Keeley, 1993
Fynboss plant species	Seed germination increased	Brown, 1993
Restionaceae family species	Seed germination increased	Brown et al., 1994
Western Australian plants	Seed germination increased	Dixon et., 1995, Tieu et al., 1999
California chaparral plants	Seed germination increased	Flematti et al., 2004
Whispering bells	Seed dormancy released	Keeley and Fotheringham 1998
Plants from Cape floristic regions	Seed germination increased	Brown, N.A.C.; Botha, 2004
12 Poaceae species	Seed germination increased	Clarke and French, 2005
Australian Asteraceae	Seed germination	Merritt et al., 2006
12 eastern Mediterranean basin plants	Seed germination increased	Catav et al., 2015
10 Interior West Penstemon species	Seed germination increased	Fornwalt, 2015
Mediterranean Basin flora	Seedling emergence was promoted	Tormo et al., 2013
Cape flats sand Fynbos species	Seedling length and seed germination were promoted	Mukundamago et al., 2017
Whispering bells	Germination percentage increased	Keeley and Fotheringham, 1997
Red oat grass	Germination percentage increased	Baxter and van Staden, 1994, Baxter et al., 1994
Stuart's heath	Germination percentage increased	Keith, 1997
Rice	Seed dormancy was released	Doherty and Cohn, 2000
Common oak, Pyrenean oak, Holly oak	Germination percentage was increased	Reyes and Casal, 2006a
Brown stringy bark, Tasman Heath	Germination percentage increased	Enright and Kintrup, 2001, Gilmour et al., 2000
Pinnate goodenia	Germination percentage increased	Sugimato and Lidbetter, 2002
African juniper	Germination percentage increased	Tigabu et al., 2007
Princess tree	Germination percentage and radical length increased	Todorovic et al., 2005
Common geranium	Somatic embryogenesis enhanced	Senaratna et al., 1999
Canola	Germination percentage and rate, seedling length and weight, plant height and leaf area increased	Abdollah, 2012
Cape snow., Dekriet	Germination percentage increased	Brown et al., 1998
Society garlic., Wild garlic	Seed germination, seedling mass, root length and root number increased	Aremu et al., 2014
Dyer's woad	Seedling mass was increased	Zhou et al., 2011
Soil seed bank species from Sydney	Germination percentage increased	Thomas et al., 2003
Edible banana	Seedling length, seedling mass, number of shoots, number of roots, number of leaves, leaf area increased	Aremu et al., 2012b
Wild oat	Seed germination increased; seed dormancy was broken	Adikins and Peters, 2001, Kepczynski et al., 2006
Coyote tobacco	Germination percentage increased	Schwachtje and Baldwin, 2004
Coyote tobacco., Whispering bells	Germination percentage increased	Preston et al., 2014
Rock rose	Germination percentage increased	Tavsanoglu, 2011
Mountain tea., Honeybush Tea	Germination percentage increased	Sutcliffe and Whitehead, 1995

Sweet potato	Number of adventitious roots, length of adventitious roots and length of lateral roots increased	Aslam et al., 2014
Pawpaw	Seed germination rate, seedling length and vigor, and number of leaves increased	Chumpookam et al., 2012
Canola	Plant regeneration, seedling length increased	Ghazanfaria et al., 2012
Lantana	Seed germination, germination velocity index and vigor index increased	Raizada and Raghubanshi, 2010
Millet	Seed germination, seedling length and seedling mass were enhanced	Pirzada et al., 2014
Wild oat	Germination percentage and per unit weight water content increased, coat rupturing was stimulated	Cembrowska-Lech and Kępczynski, 2017
Carrot	Seed germination, seedling length increased	Asaf et al., 2014
Barnyard grass	Germination percentage, relative root elongation, seedling length and seedling mass were promoted	Kamran et al., 2014
Canadian horseweed	Seed germination, seedling growth increased	Ren and Bai, 2016
Wheat	Germination percentage, germination index, seedling vigor index and seedling length increased	Iqbal et al., 2016
Wheat	Root/shoot length, root fresh/dry weight, shoot fresh/dry weight and leaf area were increased	Iqbal et al., 2018
Rice	Root length and root fresh/dry weights increased	Akhtar et al., 2017
Rice	Seed water uptake and germination percentage were enhanced	Jamil et al., 2020
Tomato, Cucumber, Pot marigold, Sword lily	Seed germination percentage/rate, seedling length and fresh weight increased	Elsadek et al., 2019
Chickpea	Seed germination, seedling length and mass increased	Rehman et al., 2018
Maize	Seed germination, seedling length and mass increased	Aslam et al., 2019
Lettuce	Seed germination percentage was promoted	Gupta et al., 2019
Trimethylebutenolide analogs and butenolide solution		
Lettuce	Germination percentage increased	Posta et al., 2013
Tangle head	Germination percentage increased	Baldos et al., 2015
Lettuce	Germination percentage increased	Ghebrehiwot et al., 2013
Lettuce., Whispering bells., Tomato bush	Germination percentage increased	Flematti et al., 2010
Smoke and butenolide solution		
Bitter aloe	Germination percentage increased	Bairu et al., 2009
Tree aloe	Seed germination and seedling growth increased	Kulkarni et al., 2013
Lettuce	Substitute for light, seed germination, seedling length and seedling mass increased	van Staden et al., 1995

Tropical soda apple	Seed germination, seedling length and mass increased	Kandari et al., 2011
Asian mustard	Germination percentage increased	Long et al., 2010
Wild oat, Wimmera ryegrass, Weeping lovegrass, Little seed canary grass, Barley grass, Perrenial veldgrass, Ripgut brome	Germination percentage increased	Long et al., 2011
Edible banana	Leaves number, branching, seedling length, seedling weight and root number increased	Aremu et al., 2012
onion	Number of leaves, leaf length, leaf weight, bulb diameter and bulb weight increased	Kulkarni et al., 2010
Okra, Common bean	Seedling mass, seedling length and leaf number increased	van Staden et al., 2006
Tomato	Seed germination, seedling length and radical emergence increased	Jain, N and van Staden, 2007
Rice	Seed germination, seedling weight, number of lateral roots and vigor index increased	Kulkarni et al., 2006a
Pineapple flower	Germination percentage increased	Kulkarni et al., 2006b
Candle thorn, Ankle thorn, Black wattle	Seedling mass increased	Kulkarni et al., 2007a
Wild yam	Seed germination and seedling vigor index increased	Kulkarni et al., 2007b
Tomato	Stem thickness, leaves number and plant height increased	Kulkarni et al., 2008
Tomato	Seed germination, seedling weight, root length, vigor index, radicle emergence and hypocotyl length increased	Jain et al., 2006
**Soybean,** Black gram	Roots imitation and callus formation were stimulated	Jain et al., 2008a
Tomato	Root length and moisture content increased	Jain et al., 2008b
Maritime pine, Scots pine, Austrian pine, Mountain pine	Germination percentage increased	Reyes and Casal, 2006b
Melon	Seedling mass increased	Mavi et al., 2010
Torch lily, Opal flower	Pollen germination and pollen tube growth was enhanced	Kumari et al., 2015
Button creeper	Seed germination was increased, and seed dormancy was broken	Downes et al., 2010
Tassel rope-rush	Seed germination and somatic embryogenesis were enhanced	Ma et al., 2006
Lettuce	Seed germination and radicle length increased	Ren et al., 2017
Kikuyu grass	Seedling vigor, seedling mass, and leaf number increased	Okem et al., 2015
Okra	Seedling length was increased	Papenfus et al., 2015
Glyceronitrile and smoke/butanolide solution		
Kangaroo paw	Seed germination and embryo growth were increased	Ma et al., 2018
Kangaroo paw., *Gyrostemon, Racemigerus.*, *Gyrostemon ramulosus*	Seed germination and seedling length were enhanced	Downes et al., 2013
Aerosol smoke and smoke solution		

Chebulic myrobalan., Kurchi, Asian pigeonwings., Gurmar	Germination percentage, vigor index and seedling height increased; seedling survival and health were good	Maitbadil and Kumar, 2006
Arabidopsis	Seed germination and hypocotyl length was increased; and seed dormancy was broken	Nelson et al., 2009, Nelson et al., 2010, Nelson et al., 2011
Foliar applications of smoke and butenolide solution		
Okra, Tomato	Seedling mass, number of leaves, total leaf area and stem thickness were increased	Kulkarni et al., 2007a
Smoke and PGPR solution		
Rice	Seed germination and shoot/root lengths were increased	Khan et al., 2017
